# Magnitude and temporal trends of leprosy relapse in the state of Ceará, Brazil in the period 2001-2018

**DOI:** 10.1590/0037-8682-0389-2020

**Published:** 2021-02-26

**Authors:** Reagan Nzundu Boigny, Caroline Mary Gurgel Dias Florêncio, Kellyn Kessiene de Sousa Cavalcante, Jarier de Oliveira Moreno, Pedro José de Almeida, Jardel Gonçalves de Sousa Almondes, Paula Sacha Frota Nogueira, Carlos Henrique Alencar

**Affiliations:** 1 Universidade Federal do Ceará, Faculdade de Medicina, Programa de Pós-Graduação em Saúde Pública, Fortaleza, CE, Brasil.; 2 Universidade Federal do Ceará, Faculdade de Farmácia, Odontologia e Enfermagem, Departamento de Enfermagem, Fortaleza, CE, Brasil.; 3 Universidade Federal do Ceará, Faculdade de Medicina, Programa de Pós-Graduação em Patologia, Fortaleza, CE, Brasil.

**Keywords:** Leprosy, Epidemiology, Cross-sectional, Recurrence

## Abstract

**INTRODUCTION:**

This study analyzed the magnitude and temporal trends of leprosy relapse in Ceará in 2001-2018.

**METHODS:**

Descriptive cross-sectional and ecological-time trend studies were performed.

**RESULTS:**

We diagnosed 1,777 leprosy relapse cases. Higher prevalence of relapse was observed in men, illiterates, mixed race, multibacillary leprosy, lepromatous leprosy, and persons with visible disabilities. The proportion of relapse increased throughout the study period.

**CONCLUSIONS:**

Leprosy relapse is prevalent in certain groups.

Leprosy is a chronic infectious disease caused by *Mycobacterium leprae*, and humans are its main host. It primarily affects peripheral nerves, skin, and mucous membranes and, when left untreated, it can lead to physical disabilities, with economic, social, and psychological impacts^1^ ^,^ ^2^ ^,^ ^3^. 

On the contrary, even if properly treated, leprosy patients can manifest reactivation of the disease; this event is described as relapse^4^ ^,^ ^5^. The Brazilian Ministry of Health considers as relapse any cases of leprosy treated regularly with standardized and correctly indicated official regimens that present new clinical manifestations of active infectious disease, usually after five years of discharge due to cure^4^. Data from the World Health Organization revealed that in 2018, there were 3,361 cases of leprosy relapse worldwide, with a proportion of 1.6% among new cases; of these, 56.2% were in Brazil. In 2019, there were 1,840 cases of relapse in Brazil, equivalent to almost 55% of all global relapse cases and a proportion of 6.4% among the new cases; however, most countries do not report this data^2^ ^,^ ^6^. 

In recent years, several studies have reported an increase in leprosy relapse cases in the Brazilian population, which can lead to physical disabilities and social isolation^7^ ^,^ ^8^. In the state of Ceará, the proportion of relapse cases increased from 3.1% in 2008 to 8.7% in 2017^2^. Thus, we aimed to analyze the magnitude and time trend of leprosy relapse cases in the state of Ceará between 2001 and 2018. 

We conducted a cross-sectional study and an ecological temporal trend study using secondary data of leprosy relapse cases in Ceará reported in the Notifiable Diseases Information System (*Sistema de Informação de Agravos de Notificação*; SINAN). The state of Ceará is in the northeast region of Brazil. It has an estimated population of approximately nine million inhabitants and a demographic density of 56.7 inhabitants per km²^,^ ^9^. 

Data were organized and analyzed using the Stata 15.1 software (StataCorp LLC, College Station, TX, USA). Descriptive data were presented using tables with absolute and relative frequencies. The presence of relapse was used as the outcome to calculate the prevalence ratios (PR) and their respective 95% confidence intervals (CI). The significance level was set at 0.05. 

The proportion of leprosy relapse cases per year was calculated by dividing the number of reported relapse cases by the total number of leprosy reported cases in that year multiplied by 100. The calculation of this proportion was standardized by age, using the direct method to allow comparisons between years. 

For trend analysis, we calculated the annual percent change (APC) and the average annual percentage change (AAPC) of the proportion of leprosy relapse using a joinpoint regression model generated by the Joinpoint Regression Program, version 4.8.0.1. This analysis uses an algorithm that tests whether a multisegment line is significantly better than a single line or a line with fewer segments^10^. 

The joinpoint regression analysis joins a series of straight lines on a logarithmic scale to detect the trend of the annual value of the indicator. Each joinpoint indicates a change in the trend of the indicator^10^. The Monte Carlo permutation test was used for determining statistical significance, which chooses the best number of segments for each model. 

We considered a model as statistically significant if it displayed an estimated p-value <0.05. To perform the joinpoint analysis, we used the number of relapses as the numerator and the number of new cases as denominator, and multiplied the divided value by 100. We performed a logarithmic transformation of the data. 

The errors were considered heteroscedastic, and the regression coefficients were estimated by weighted least squares. Considering this and the temporal evaluation of the data, an adjusted model of autocorrelation of the errors based on the data was also employed. 

The CIs of APC and AAPC were based on the *t* distribution and the empirical quantile method, respectively. This method generates resampled data by (i) generating resampled residuals as the inverse function values of the uniform random numbers over (0, 1), where the function is the empirical distribution function of the original residuals and then (ii) adding resampled residuals to the original fit. A total of 10,000 resamples were used in our analyses. Lastly, we considered a maximum of three joinpoints for the study period.

Of the 41,759 reported leprosy cases, there were 1,777 (4.3%) cases of leprosy relapse. Relapse cases were predominant in males (1,157 cases; 65.1%), followed by individuals belonging to mixed race (908 cases; 51.1%), with elementary education (742 cases; 41.8%), and in the age groups of the economically active population: 30 to 39 years (311 cases; 17.5%), 40 to 49 years (369 cases; 20.8%), and 50 to 59 years (409 cases; 23.0%). 

Clinically, the borderline (691 cases; 38.9%) and lepromatous (535 cases; 30.1%) clinical forms were predominant as well as the multibacillary classification (1,543 cases; 86.8%). We classified 774 cases (43.6%) as having no physical disabilities (grade 0); however, the majority had positive bacilloscopic examination (496 cases; 49.7%) (Table 1). 

**TABLE 1:** Association of sociodemographic and clinical factors to leprosy relapse cases in the state of Ceará (2001-2018).

Variables	Total	Relapse		PR	95% CI
		n	%		
**Ano**					
2001	2663	84	3.1	Ref	
2002	2524	87	3.5	1.09	0.81-1.47
2003	2852	106	3.7	1.18	0.89-1.56
2004	2724	101	3.7	1.17	0.88-1.56
2005	2796	93	3.3	1.05	0.79-1.41
2006	2486	89	3.6	1.13	0.85-1.52
2007	2532	74	2.9	0.93	0.68-1.26
2008	2608	81	3.1	0.98	0.73-1.33
2009	2336	78	3.3	1.06	0.78-1.43
2010	2231	89	4.0	1.26	0.94-1.69
2011	2084	97	4.6	1.47	1.11-1.96
2012	2196	88	4.0	1.27	0.94-1.70
2013	2164	111	5.2	1.63	1.23-2.15
2014	2139	112	5.2	1.66	1.26-2.19
2015	1913	100	5.2	1.66	1.25-2.20
2016	1824	122	6.7	2.12	1.62-2.78
2017	1678	128	7.6	2.42	1.85-3.16
2018	1821	137	7.5	2.38	1.83-3.11
**Sex**					
Feminine	19096	620	3.3	Ref	
Male	22475	1157	5.2	1.58	1.44 -1.74
Race/color*					
White	7151	262	3.7	Ref	
Black	2957	165	5.6	1.52	1.26-1.84
Yellow	422	12	2.8	0.77	0.44-1.37
Brown	19462	908	4.7	1.27	1.11-1.46
Indigenous	93	7	7.5	2.05	0.99-4.23
Education level*					
Illiterate	6344	292	4.6	1.99	1.30-3.06
Elementary school	17975	742	4.1	1.79	1.17-2.72
High school	4264	162	3.8	1.64	1.06-2.56
Higher education	955	22	2.3	Ref	
**Age range**					
under 15	2034	15	0.4	Ref	
15 to 19 years	1859	29	1.6	2.11	1.14-3.93
20 to 29 years	4848	161	3.3	4.50	2.66-7.62
30 to 39 years	6329	311	4.9	6.66	3.97-11.16
40 to 49 years	7620	369	4.8	6.59	3.94-11.03
50 to 59 years	7643	409	5.4	7.26	4.34-12.11
60 to 69 years	5985	283	4.7	6.41	3.82-10.75
70 to 79 years	3648	145	4.0	5.39	3.18-9.15
80 years or older	1605	55	3.4	4.65	2.63-8.19
Clinical form***					
Indeterminate	5665	129	2.3	1.49	1.17-1.89
Tuberculoid	8812	134	1.5	Ref	
Borderline	14545	691	4.8	3.03	2.52-3.64
Lepromatous	7319	535	7.3	4.55	3.77-5.48
Physical disability grade during diagnosis**					
Grade 0	25322	774	3.1	Ref	
Grade 1	8142	442	5.4	1.74	1.55-1.95
Grade 2	2861	246	8.6	2.67	2.32-3.07
**Operational classification**					
Paucibacillary	15848	229	1.4	Ref	
Multibacillary	25653	1543	6.0	4.16	3.63-4.77
Bacilloscopy***					
Positive	8157	581	7.1	2.10	1.83-2.40
Negative	9128	299	3.3	Ref	

**PR:** prevalence ratios; **CI**: confidence intervals.

95% CI that does not contain a value of 1 suggests significant associations (similar to p<0.05).

*Ignored; **Not classified; ***Ignored and not classified.

Race/color: 11471 ignored; Education level: 12033 ignored; Clinical form: 49 ignored and 283 not classified; Physical disability grade: 78 ignored and 307 not classified; Operational classification: 70 ignored; Bacilloscopy: 21030 ignored and 152 not classified.

The magnitude of relapses ranged from 74 (2.9%) cases in 2007 to 137 (7.5%) in 2018. Between 2001 and 2004, the number of relapses increased from 84 (3.1%) to 101 (3.7%) cases, remaining almost similar in 2005 (93; 3.3%) and 2006 (99; 3.6%). The number of relapses increased gradually, reaching 128 (7.6%) and 137 (7.5%) cases in 2017 and 2018, respectively. After 2013, we observed 1.5 times higher and statistically significant prevalence rates. 

The prevalence of relapse was significantly higher among men (PR = 1.58; 95% CI: 1.44-1.74), self-declared black people (PR = 1.52; 95% CI: 1.26-1.84), and mixed race individuals (PR = 1.27; 95% CI: 1.11-1.46). Illiterate people (PR = 1.99; 95% CI: 1.30-3.06) and those with elementary (PR = 1.79; 95% CI: 1.17-2.72) or high school (PR = 1.64; 95 %CI: 1.06-2.56) education were also associated with relapse. The prevalence of relapse increased progressively with age (Table 1). 

Relapse was associated with the indeterminate (PR = 1.49; 95% CI: 1.17-1.89), borderline (PR = 3.03; 95% CI: 2.52-3,64), and lepromatous (PR = 4.55; 95% CI: 3.77-5.48) clinical forms of leprosy. Similarly, the prevalence of relapse was higher among multibacillary leprosy cases (PR = 4.16; 95% CI: 3.63-4.77). 

The prevalence of relapse was higher in patients with physical disabilities of both grade 1 (PR = 1.74; 95% CI: 1.55-1.95) and grade 2 (PR = 2.67; 95% CI: 2.32-3,07) as well as in those with positive bacilloscopic examination (PR = 2.10; 95% CI: 1.83-2.40). 

We observed two different trends-a decreasing but not significant trend from 2001 to 2008 (APC = −1.5; 95% CI: −5.2-2.3) and an increasing and statistically significant trend from 2008 to 2018 (APC = 9.6; 95% CI: 7.2-12.1). However, the proportion of relapse tended to increase significantly throughout the study period (AAPC = 4.9; 95% CI: 3.1-6.9) (Figure 1; Table 2).

**FIGURE 1:**
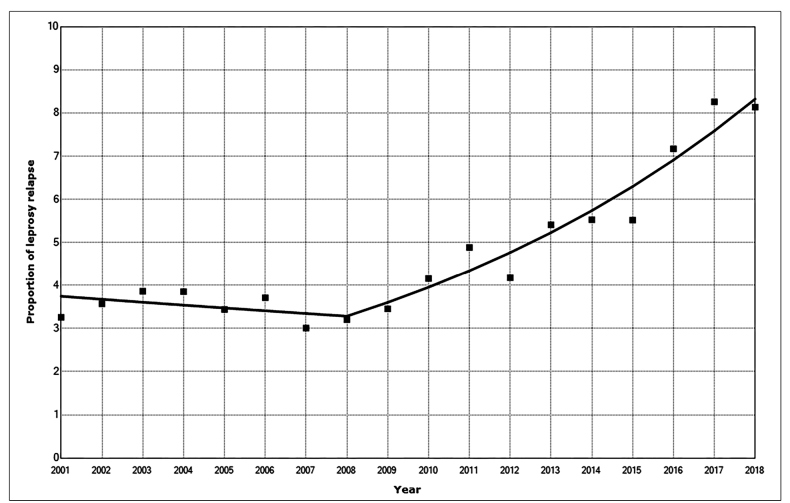
Temporal trends of leprosy relapse in Ceará, Brazil between 2001 and 2018 (The solid squares are the observed values and the line the predict ones).

**TABLE 2:** Time trend in the proportion of leprosy relapse in the state of Ceará, Brazil (2001-2018).

Group	Period	AAPC	APC	95% CI
Total cases	2001 to 2018	4.9		3.1-6.9
	2001 to 2008		−1.5	−5.2-2.3
	2008 to 2018		9.6	7.2-12.1
Male	2001 to 2018	5.3		3.7-7.2
	2001 to 2009		−0.4	−3.3-2.6
	2009 to 2018		10.7	8.5-13.1
Female	2001 to 2018	3.5		0.0-7.2
	2001 to 2007		−3.4	−11.4-5.4
	2007 to 2018		7.4	3.7-11.3
20 to 29 years	2001 to 2018	7.9		2.5-13.5
	2001 to 2010		2.6	−4.6-10.3
	2010 to 2018		14.1	4.6-24.4
30 to 39 years	2001 to 2018	5		0.2-10.0
	2001 to 2012		0.9	−3.7-5.8
	2012 to 2018		12.9	0.3-27.1
40 to 49 years	2001 to 2018	7		1.1-14.6
	2001 to 2010		0.4	−8.9-10.6
	2010 to 2018		15.1	2.5-29.2
50 to 59 years	2001 to 2018	1.2		−6.3-9.2
	2001 to 2003		−36.4	−67.9-25.9
	2003 to 2018		7.6	4.5-10.8
60 to 69 years	2001 to 2018	7.3		−3.8-19.7
	2001 to 2016		4.1	−0.1-8.5
	2016 to 2018		34.2	−49.5-256.2
70 to 79 years	2001 to 2018	2.9		−3.7-10.1
	2001 to 2007		−5.7	−20.2-11.5
	2007 to 2018		8	1.1-15.4
80 years or older	2001 to 2018	1.2		−9.5-13.2
	2001 to 2009		−13.8	−28.8-4.4
	2009 to 2018		16.7	−0.6-37.1

**AAPC:** average annual percentage change; **APC:** annual percent change; **CI:** confidence intervals.

95% CI that does not contain a value of 0 suggests significant associations (similar to p<0.05).

The proportion of relapses in men showed an increasing trend over the study period (AAPC = 5.3; 95% CI: 3.7-7.2); however, between 2001 and 2009, the trend decreased (APC = −0.4; 95% CI: −3.3-2.6). In the female population, the trend was upward (AAPC = 3.5; 95% CI: 0.0-7.2) but without statistical significance (Table 2). 

The proportion of relapse significantly increased in the age groups of the economically active population. This trend was higher in the age groups 20 to 29 years (AAPC = 7.9; 95% CI: 2.5-13.5) and 40 to 49 years (AAPC = 7.0; 95% CI: 1.1-14.6). In older patients, the proportion of relapse tended to increase during the entire period but without statistical significance. The age group of 70 to 79 years was the only one that showed a significant increasing trend between 2007 and 2018 (APC = 8; 95% CI: 1.1-15.4) (Table 2). 

The present study reveals that leprosy relapse in the state of Ceará in Brazil is a growing phenomenon in almost all age groups and in both sexes. We found a significant association between the sociodemographic and clinical variables analyzed. The finding of higher prevalence in males is consistent with other studies, emphasizing the importance of monitoring leprosy relapse in men^11^ ^,^ ^12^. 

The fact that relapse is significantly associated with mixed and black races runs through the dimension of the health sphere; this exhibits different situations of vulnerability, both social and programmatic^13^ ^,^ ^14^. The state of social vulnerability experienced by these cases can result in their nonadherence to treatment. 

As for programmatic vulnerability, black and mixed-race people in Brazil generally have more restricted access to the healthcare system or, when they do, the care provided is often low quality^13^ ^,^ ^14^. This fact is aggravated when they are affected by infectious and neglected diseases, such as leprosy, that carry strong social stigma^13^ ^,^ ^14^. However, in a study conducted in the state of Espírito Santo, the prevalence of leprosy relapse was higher in self-reported white groups^15^. 

Education was also associated with relapse, corroborating with other studies that demonstrated lower level of education in most cases of leprosy^7^ ^,^ ^12^ ^,^ ^14^ ^,^ ^15^. The increase in prevalence of relapse in the economically-active population could be explained by the long incubation period of the disease^11^ ^,^ ^12^ ^,^ ^14^ ^,^ ^15^. 

Additionally, leprosy relapses were associated with indeterminate, borderline, and lepromatous clinical forms of leprosy, consistent with the results of the research conducted between 2005 and 2007 in the state of Mato Grosso^7^. The prevalence of relapse was higher among multibacillary leprosy cases, with some physical disabilities and positive bacilloscopic exam at the time of diagnosis. Studies revealed that multibacillary leprosy patients are at higher risk of developing relapse compared to the paucibacillary leprosy patients due to high bacillary load^7^ ^,^ ^12^ ^,^ ^15^. However, some patients initially classified as paucibacillary in the diagnosis of relapse changed their classification to multibacillary^15^. Serological tests can assist in the correct classification, emphasizing that the occurrence of error could be associated with the unpreparedness of the healthcare workers^7^ ^,^ ^11^ ^,^ ^12^. A significant proportion of cases with relapse that did not undergo an assessment of the physical disability at the time of diagnosis is worrying, demonstrating the limitation of the healthcare professionals to correctly evaluate these cases, in addition to the quality of the assistance offered. 

The use of secondary data is a limitation of this research, as it made it impossible to use other variables. To minimize these inconsistencies, incorrect or missing information were excluded from the analysis. 

In conclusion, our study revealed that the burden of leprosy relapse in the state of Ceará in Brazil is concentrated in the male population, self-declared black and mixed-race people, economically-active age groups, and individuals with elementary education. Multibacillary relapse cases were prevalent in addition to some physical disabilities during diagnosis and a positive bacilloscopic examination. The time trend increased throughout the study period. These findings may assist healthcare services developing policies and strategies to prevent leprosy relapses and to cease its transmission in the state of Ceará.
